# Reviews

**Published:** 1880-10

**Authors:** 


### REVIEWS.

The report of Dr. James Law, of Ithaca, N. Y., on the contagious diseases and parasites of animals, published in the Bulletin of the National Board of Health, Vol. 2, No. 4, merits more than usual attention. He gives in it a list of diseases and parasites which may be communicated from animals to man and from animals to each other, which we below append, and in doing so, suggest that three others be credited to the first section, viz.: Scarlet fever in the Felida and pertussis and catarrhal conjunctivitis in the Canine. It shows very conclusively by a simple arrangement of facts that the sanitary condition of our domestic animals and birds should be looked into by those who are qualified to judge, as well as that of human beings, to insure a greater degree of public health. Since both animals and birds are subject to these contagious diseases, the epidemics of such nature in isolated and distant places, should and cannot be any longer enveloped in mystery.

Glanders, hydrophobia, anthrax, and vaccine have long been known to be communicable from animals to men, but many others of the list are of recent discovery. The same is likewise true of many of the parasitic plagues.

*A—Contagia common to Man and Animals.*

1. Glanders and farcy in horses, etc.
2. Canine madness, rabies in dogs, cats, etc.
3. Malignant anthrax in all domestic animals.
4. Tuberculosis in all animals.
5. Asiatic (malignant) cholera in all animals.
6. Milk-sickness in cows and other animals.
7. Small pox in chickens, pigeons, etc.
8. Eczematous (aphthous) fever in bisulcates, etc.
9. Typhoid fever (?) in sucking animals.
10. Diphtheria in animals.

*B—Parasites common to Man and Animals.*

1. Echinococcus in animals: Tænia echinococcus in dogs.
2. Cysticercus cellulosa in swine: Tænia solium in man.
3. C. medio-cannellata in calves: T. medio-cannellata in man.
4. C. tenuicollis in man, sheep, etc.: T. marginata in dogs.
5. Tænia elliptica in man and cat.
6. Bothriocephalus latus in man, dog, etc.
7. B. cordatus in man, dog, etc
8. Trichina spiralis in swine, etc.
9. Tricocephalus dispar in man and pig.
10. Strongylos gigas in man, horse, ox, and dog.

11. Ascaris mystax in cat and human being.
12. Fasciola hepatica in man, herbivora, and omnivora.
13. Distomum lanceolatum in man, herbivora, and omnivora.
14. Pentastoma taenioides in man, dog, sheep, etc.
15. Sarcoptis mutans in chickens and man.
16. Demodex folliculorum in dog, sheep and man.
17. Æstrus bovis and other cuticolla in cattle and man.
18. Gregarina in man and animals.
19. Tricophyton tousurans in man and animal (Tinea tonsurans.)
20. Achorion schönleini in man and animals (Tinea favosa.)
21. Microsporon adonini in man and animals (Tinea decalvans.)
22. Oidium albicans in man and animals (Thrush. muguet.)

*C—Contagia communicable from one animal to another.*

1. Texas fever in cattle.
2. Swine plague. Intestinal fever of swine. Hog cholera.
3. Bovine lung plague. Contagious pleuro-pneumonia in cattle.
4. Rhinderpest in cattle and other ruminants.
5. Sheep pox. Variola ovina.
6. Swine-pox. V. suilla.
7. Cow-pox. Horse-pox.
8. Venereal diseases of stallions. Dourine.
9. Foot-rot in sheep.
10. Strangles in horses.
11. Influenza in animals.
12. Infectious mammitis in cows.
13. Parturition fever in ewes.
14. Quebra bunda in horses.
15. Horse sickness of South Africa.

*D—Parasites causing enzootics in animals.*

1. Scabies acariasis:

   - In sheep

     - Follicular scabies. Demodex folliculorum.

     - Scab

       - Dermatocoptis ovis.

       - Dermatophagus ovis.

     - Black nose. Sarcoptis ovis, (Leptus Americani ?)

   - In horse

     - Mange

       - Sarcoptus equi.

       - Dermatocoptis equi.

     - Poultry mange. Dermanyssus avium, etc.

     - Foot mange. Dermatophagus equi

   - In ox.....

     - Mange. Dermatocoptis bovis.

     - Foot mange. Dermatophagus bovis.

     - Poultry mange. Dermanyssus avium, etc.

   - In pigs and dogs, mange. Sarcoptis suis, (Squamiferous.)

   - In dog...

     - Mange. Sarcoptis squamiferous, (Suis.)

     - Follicular mange. Demodex folliculorum.

In cat and rabbit, mange. Sarcoptis nivar.

In chicken, scabies... { Sarcoptis mutans. / Dermanyssus avium.

2. Lung worms:

In sheep, goat, and camel { Strongylus filaria. / Strongylus filaria, varietas longus. / Strongylus Africana.

In horse and ox, Strongylus micrurus.

In swine, Strongylus elongatus.

In birds, gapes. Sclerostomum syngamus.

3. Selerostomum hy ostomum in sheep.
4. Strongylus filicollis in sheep.
5. Strongylus contortus in sheep and cattle
6. Sclerostomum equinum in horse.
7. S. tetracanthum in horse.
8. S. suis in swine.
9. Tricocephalus affinis in cattle and sheep.
10. Stephanurus dentata in swine.
11 Echinorynchus gigas in swine, cockroach, ladybug, etc.
12. Ascaris suilla in swine.
13. Tænia expansa in sheep and cattle.
14. Cœnurus cerebralis in sheep, cattle, dogs, etc.
15. Œstrus ovis, grub in the head, in sheep.

---

The a ticle by George Fleming, F. R. C. V. S., on Tuberculosis from a sanitary and pathological point of view published in the *Veterinary Journal* and concluded in the August number, is full of important facts for every reader to consider.

He arrays old, well-established truths in new forms, and strengthens them by his own experience and the experience of other observers of the highest character; also gives many startling developments, the results of late experiments and observation, such as the positive communication of tuberculosis from animal to animal of the same and of different species, and from animal to man, and the character and condition by which it may be done.

---

The National *Live Stock Journal* of Chicago, an able and admirable Journal, contains in its September number a most sensible article on *the cattle business* of the *western plains.* In this article the editor deals in truth, and not in fiction; in facts, and not in theory.

